# External validation of the MAGNIFI-CD index in patients with complex perianal fistulising Crohn’s disease

**DOI:** 10.1007/s00330-024-11029-3

**Published:** 2024-08-30

**Authors:** Kim J. Beek, Lieven G. M. Mulders, Kyra L. van Rijn, Karin Horsthuis, Jeroen A. W. Tielbeek, Christianne J. Buskens, Geert R. D’Haens, Krisztina B. Gecse, Jaap Stoker

**Affiliations:** 1 Department of Radiology and Nuclear Medicine, Amsterdam UMC, Location University of Amsterdam, Meibergdreef 9, Amsterdam, The Netherlands; 2 Amsterdam Gastroenterology Endocrinology Metabolism, Amsterdam, The Netherlands; 3 Department of Gastroenterology and Hepatology, Amsterdam UMC, Location University of Amsterdam, Meibergdreef 9, Amsterdam, The Netherlands; 4 Department of Radiology and Nuclear Medicine, Amsterdam UMC, Location Vrije Universiteit, De Boelelaan 1117, Amsterdam, The Netherlands; 5 Spaarne Gasthuis, Department of Radiology and Nuclear Medicine, Boerhaavelaan 22, Haarlem, The Netherlands; 6 Department of Surgery, Amsterdam UMC, Location University of Amsterdam, Meibergdreef 9, Amsterdam, The Netherlands

**Keywords:** Crohn’s disease, Rectal fistula, Magnetic resonance imaging, Treatment outcome

## Abstract

**Background:**

There is an increasing need for objective treatment monitoring in perianal fistulising Crohn’s disease (pfCD). Therefore, the magnetic resonance novel index for fistula imaging in CD (MAGNIFI-CD) index has been designed and internally validated on the ADMIRE-CD trial cohort. The aim of this study was to externally validate the MAGNIFI-CD index to monitor response to medical and surgical treatment regimens in pfCD.

**Methods:**

A retrospective longitudinal cohort was established of consecutive patients with complex pfCD treated with surgical and/or medical therapy and a baseline and follow-up MRI between January 2007 and May 2021. The MAGNIFI-CD index was scored by two independent, abdominal radiologists blinded for time points and clinical outcomes. Responsiveness, reliability, and test accuracy regarding clinically important improvement were assessed. Cut-offs for response and remission were selected classified on fistula drainage assessment and physician global assessment.

**Results:**

A total of 65 patients (51% female, median age 32 years) were included. A clinically relevant responsiveness of the MAGNIFI-CD was shown, with a significant decrease in clinical remitters and responders with a median MAGNIFI-CD of 18.0 [7.5–20.0] to 9.0 [0.8–16.0] (*p* < 0.001) and non-significant change in non-responders with a median MAGNIFI-CD of 20.0 [12.0–23.0] to 18.0 [13.0–21.0] (*p* = 0.22). There was an ‘almost perfect’ interobserver agreement (ICC = 0.87; 95% CI 0.80–0.92) for the MAGNIFI-CD index. An optimal cut-off value was defined as a decrease of 2 points for clinical response, and a MAGNIFI-CD ≤ 6 for remission at follow-up MRI.

**Conclusion:**

The MAGNIFI-CD index is a responsive and reliable MRI scoring instrument for treatment monitoring in perianal fistulising Crohn’s disease.

**Clinical relevance statement:**

The MAGNIFI-CD index is a well-structured, responsive scoring instrument to assess fistula severity and activity that allows quantitative detection of changes in therapy response in patients with perianal fistulising Crohn’s disease, thereby facilitating endpoints in clinical trials.

**Key Points:**

- *Well-defined cut-offs for response and remission are needed for objective treatment monitoring of perianal fistulising Crohn’s disease (pfCD)*.
- *Cut-off values for remission and for response at 6 months follow-up were defined. Interobserver agreement was good*.
- *The MAGNIFI-CD index is responsive and reliable for treatment monitoring and is suitable for use in clinical trials*.

**Graphical Abstract:**

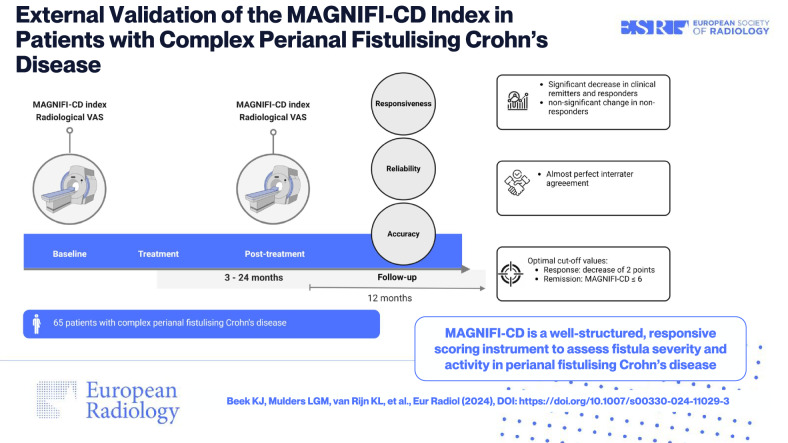

## Introduction

Perianal fistulising Crohn’s disease (pfCD) occurs in up to 30% of patients with Crohn’s disease (CD) [1]. Discharge, pain, faecal incontinence, and recurrent abscesses cause a high disease burden [2]. During the disease course, most patients require treatment that is medical, surgical, or a combination of both [3]. pfCD is challenging to treat and is often refractory to therapy and therefore up to 25% of the pfCD population with complex fistulas end up with an ostomy or ultimately a proctectomy [4–6].

In clinical trials on perianal fistulising Crohn’s disease the primary outcome has shifted from merely clinical towards combined clinical and radiological outcome [7, 8]. Radiological healing, defined as the absence of hyperintensity on T2-weighted images and no contrast uptake at fat-suppressed T1-weighted images, has been associated with long-term clinical remission [9]. Recent clinical trials used the absence of abscesses (> 2 cm) as a (combined) radiological endpoint [8], albeit a parameter of recurrence instead of therapy response and thus an underestimation of radiological fistula status and healing. However, previously developed radiological scoring systems, such as the (modified) van Assche index and the MAGNIFI-CD have not been fully validated [10, 11]. Therefore, for adequate treatment monitoring in pfCD, there is an increasing unmet need for a fully validated radiological disease activity index.

The MAGNIFI-CD index was developed based on rigorous methodology using the scans from the ADMIRE-CD trial. MRI items were selected from a list of items based on expert opinion and from the items of the (modified) van Assche index if they reached an ICC ≥ 0.40 [11, 12]. The developed MAGNIFI-CD index was internally validated regarding reliability (intra- and interobserver agreement) and responsiveness. Responsiveness was analysed by means of longitudinal validity based on correlation coefficients among change scores for the MAGNIFI-CD index with clinical and radiological change scores (i.e., radiological Visual Analogue Scale (rVAS)). In addition, standardised effect sizes were measured by means of a predefined statistically meaningful change defined as an improvement in the rVAS of one-half of the baseline standard deviation [12]. However, well-defined cut-offs for clinical response and remission and external validation are lacking.

The aim of this study was to externally validate the MAGNIFI-CD index regarding reliability, responsiveness, and test accuracy and to subsequently establish cut-off values for clinically relevant improvement.

## Materials and methods

### Patient selection

This study was performed at two tertiary IBD referral centres with a specialised fistula clinic—with aligned MRI scanning and treatment protocols—that merged during the study period (Location AMC and Location VUMC, Amsterdam UMC, The Netherlands). The study protocol was approved by the medical ethics committee AMC and informed consent was waived. Patients (≥ 16 years old) with complex pfCD with pelvic MRI before and 3–24 months after start or intensification of medical and/or surgical treatment were eligible. Complex fistulas were defined as multi-branched or high (involvement of upper two-thirds of sphincter complex) transsphincteric, extrasphincteric and suprasphincteric fistulas. Exclusion criteria were chronic setons (> 12 months), superficial or rectovaginal fistulas only, participation in the ADMIRE-CD study [8], incomplete MR protocol, unconfirmed CD diagnosis or incomplete clinical data [13].

### MR imaging

All MRIs were acquired with 1.5 Tesla (Siemens Avanto Fit) or 3.0 Tesla (Philips Alpha and Omega) scanners. The imaging protocol consisted of T2-weighted sagittal, coronal, and axial sequences (with and without fat-suppression (FS)) and axial T1-weighted images with FS after administration of gadolinium-based contrast agent (0.1 mmol/kg). Coronal and axial sequences were angulated, parallel and perpendicular to the anal canal, respectively. Patients received intravenous anti-peristaltic medication (scopolamine butylbromide, Buscopan; AS KALCEKS).

### Clinical outcomes

The clinical outcome measures were fistula drainage assessment upon gentle finger compression (FDA) [14] and a physician global assessment index (PGA) scored as remission, response and non-response (Supplementary Table 1). During follow-up, inflammatory markers (i.e., serum C-reactive protein (CRP) and faecal calprotectin), change in medication and CD-related surgery were collected. Both clinical outcomes were scored at 6 months (short-term) and at 18 months or until the patient was discharged from the outpatient clinic (long-term).

All clinical outcomes were scored independently after chart review and blinded for MRI outcomes by two clinicians (L.M. and K.J.B.). Disagreement was settled by a third independent gastroenterologist specialised in inflammatory bowel disease with more than 10 years of experience in pfCD (K.G.).

### MRI assessment

Prior to the radiological scoring, a training session was organised to train the three most difficult items (three cases per item) of the MAGNIFI-CD index (dominant feature, extension and inflammatory mass) [15] and go through the definitions of the MAGNIFI-CD items. In addition, the 100 mm radiological Visual Analogue Score for an overall weighing of total disease activity (rVAS) was explained and trained—after an expert abdominal radiologist (J.S.) formulated a reference standard (Table 1 and Supplementary Fig. 1) [12].

**Table 1:** Scoring options and definitions of the individual items of the MAGNIFI-CD index, their individual weights and calculation of the MAGNIFI-index

Item	Scoring options and definitions	Weight
Number of (active) fistula tracts	0 = None No tracts visible	3
	1 = Single, unbranched Single tracts with one internal opening	
	2 = Complex Either a single internal opening leading to more than one fistula tract or multiple internal openings	
T1-hyperintensity	0 = Absent/mild No hyperintensity visible/slight increase in signal intensity but less than nearby in-plane vessels	2
	1 = Pronounced Tract showing equal or greater signal hyperintensity than nearby in-plane vessels	
Predominant feature	0 = Predominantly fibrous > 50% of tract has a fibrotic appearance (i.e., hypointense on fat-saturated T2-weighted images)	2
	1 = Predominantly filled with granulation tissue > 50% of tract is filled with granulation tissue (i.e., hyperintense on fat-saturated T2-weighted images with enhancement of contents and wall on T1-weighted on post-contrast images)	
	2 = Predominantly filled with fluid or pus > 50% of tract is filled with fluid or pus (i.e., hyperintense on fat-saturated T2-weighted images with no enhancement of contents on fat-saturated post-contrast T1-weighted images (though rim enhancement may be present))	
Fistula length	0 = < 2.5 cm	2
	1 = 2.5–5 cm	
	2 = > 5 cm	
	Focused on the active part of the fistula tract (defined as hyperintense on fat-saturated T2-weighted images)	
Extension	0 = Absent No extension	2
	1 = Horseshoe Extends into the intersphincteric space on both sides of the midline.	
	2 = Infra/supralevatoric Extends upward in the ischioanal fossa but remains below the levator ani muscle/ Any extension in the supralevatoric space (i.e., above where the levator plate is connected to the anorectum)	
Inflammatory mass	0 = Absent No inflammatory mass	1
	1 = Focal Lesion > 3 mm in diameter on T2-weighted images (but does not include linear tract with diameter > 3 mm) with diffuse enhancement on T1-weighted post-contrast images (i.e., granulation tissue)	
	2 = Diffuse Diffuse inflammation of surrounding tissues	
	3 = Small collection Circumscribed cavity 3–10 mm in diameter (but does not include linear tracts with diameter > 3 mm). Hyperintense appearance on fat-saturated T2-weighted images with rim enhancement on T1-weighted post-contrast images	
	4 = Medium collection As defined above except diameter measures 11–20 mm	
	5 = Large collection As defined above except diameter measures > 20 mm	
Total MAGNIFI-CD		Range 0–25

Subsequently, all items of the MAGNIFI-CD index and rVAS were scored on baseline and follow-up MRIs by two independent abdominal radiologists (J.T. and K.H.) with experience (14 and 21 years) and specific interest in MRI in pfCD. In case of disagreement, a third experienced abdominal radiologist (expert in pfCD and > 30 years of experience (J.S.)) reassessed the scans and resolved the items of disagreement by choosing one of the two scores. All radiologists were blinded for clinical information and timing of the MRI scans. All MRIs were read in random order.

Indications for the follow-up MRIs were recorded and scored as either proactive (i.e., in case of treatment evaluation) or reactive (i.e., clinical suspicion of recurrence or abscess and patient-reported symptoms).

### Statistical analysis

All data were reported as median with interquartile range (IQR), mean with standard deviation (SD) or percentages (%) of the total cohort when appropriate. Responsiveness of the MAGNIFI-CD was analysed for (1) radiologically meaningful change (in Hindryckx et al indicated as predefined statistical meaningful change [12]), defined as an improvement in the rVAS of one-half of the baseline standard deviation (SD), and (2) clinically relevant change for clinical remitters, responders and non-responders. Results to detect a radiologically meaningful change were reported in standardised effect size according to Cohen’s d (mean difference divided by pooled SD); the interpretation was done according to Cohen’s benchmarks [16]. The MAGNIFI-CD index at baseline and follow-up for a radiologically meaningful change was reported in mean—according to Hindryckx et al [12]—and median (according to normality). Wilcoxon signed rank test based on normality was used to assess change in MAGNIFI-CD over time. Correlations coefficients for change scores of the MAGNIFI-CD index and rVAS, CRP and faecal calprotectin were analysed with either a Pearson’s or Spearman’s correlation test based on normality and interpreted according to Schober et al [17].

Reliability was analysed by means of a Cohen (quadratic) weighted kappa (κ) statistic for ordinal variables and intraclass correlation coefficients (ICC) with a two-way random model (absolute agreement) for continuous variables. Values were interpreted according to Landis and Koch [18].

To define clinical response and remission, different cut-off values in MAGNIFI-CD were evaluated. Clinical response and remission, according to the FDA and PGA, were used as binary classifier. ROC curves were constructed for (1) MAGNIFI-CD at follow-up, (2) absolute improvement in MAGNIFI-CD and (3) relative improvement in MAGNIFI-CD. For a series of cut-off values for MAGNIFI-CD at follow-up (20 to 1), absolute improvement (> 0 to > 10) and relative improvement (> 20% to 100%) sensitivity, specificity, positive and negative predictive values (PPV/NPV), Youden indices (YI) and odds ratios (OR) were calculated. Absolute cut-off values for MAGNIFI-CD at follow-up were only calculated in patients with a baseline value higher than the cut-off [19]. Cut-offs were selected to determine clinical remission (MAGNIFI-CD at follow-up) and clinical response (absolute and relative improvement), based on the highest YI and OR. The selected cut-offs were subsequently evaluated in combinations. The association between clinical response parameters and the cut-offs were determined with Pearson χ^2^.

A *p*-value of < 0.05 was considered statistically significant. Statistical analyses were performed using SPSS version 28 (IBM Corp.).

### Sample size justification

Given the retrospective design of our study, where we extracted all eligible patients from existing records, the sample size estimation was not driven by the typical prospective study design constraints. However, to justify the sample size we made estimations for the analyses for reliability and responsiveness. For reliability, a sample size of 41 patients would have at least 80% power to detect an ICC of 0.74 (based on previously reported inter-rater reliability of 0.74 by Hindryckx et al [12]) under the null hypothesis of 0.5 with two readers using an F-test with a significance level of 0.05. Sample size calculation for responsiveness was based on the estimation of standardised effect size as previously reported [12]. Specifically, a sample size of ten patients—in the group of patients with a rVAS improvement of one-half of baseline SD—would be needed to evaluate responsiveness and have 80% power to detect an effect size of 1.02 using a paired *t*-test with a 5% two-sided significance level. Because the exploration of optimal cut-off values was intended to be hypothesis generating, the results of statistical testing in the paragraph on test accuracy should be interpreted as assisting in identifying a potential association rather than confirming a hypothesis. Power estimations were calculated using nQuery version 8 (Statsols).

## Results

### Patient selection

**Fig. 1:**
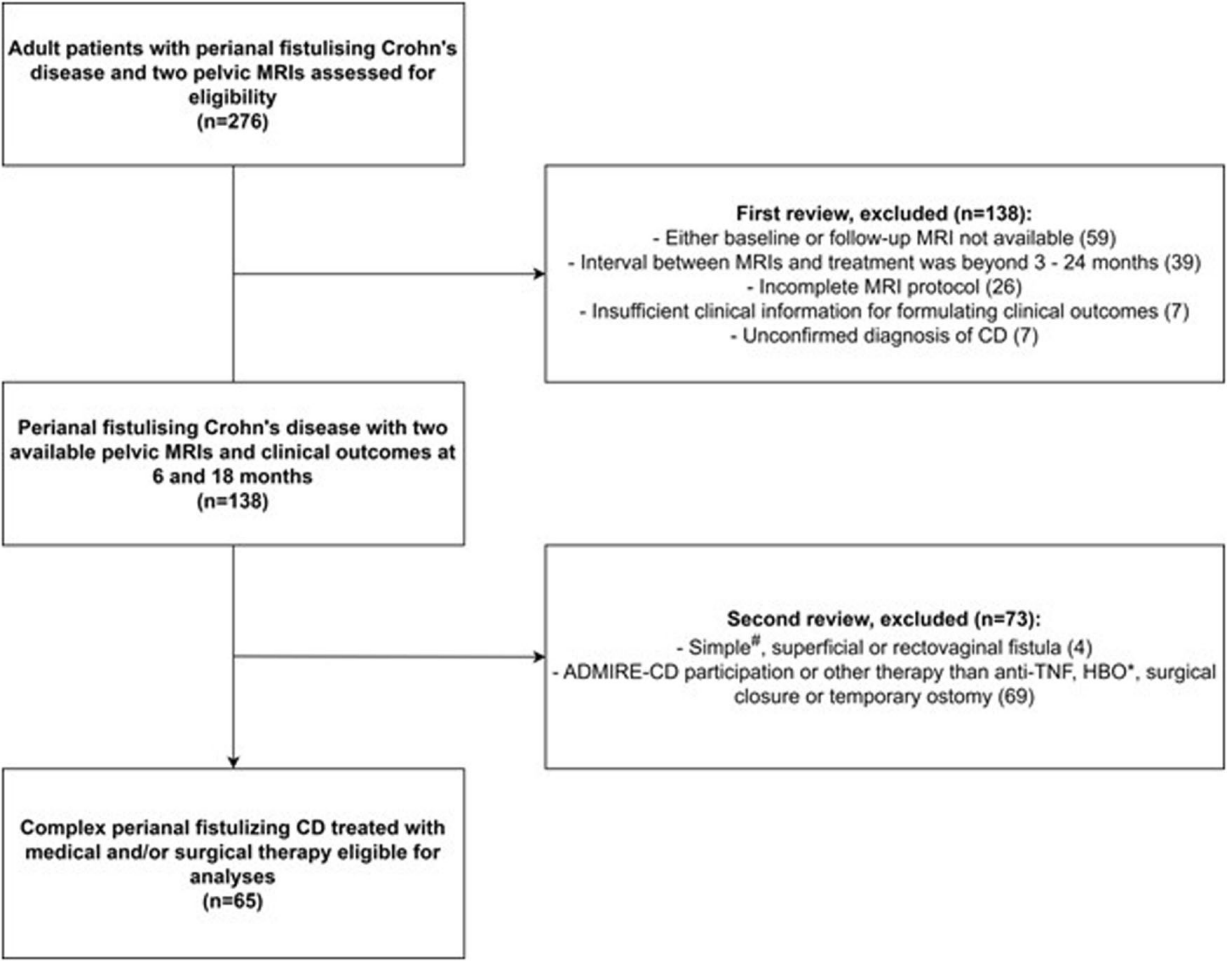
Patient flowchart ^#^Simple fistula is defined as a single tract without branching, affecting less than one-third of the sphincter complex (low), without proctitis or an abscess [23]. *HBO, hyperbaric oxygen therapy

An electronic database search yielded 276 cases of patients with pfCD who had undergone two subsequent pelvic MRIs. After applying the in- and exclusion criteria, 65 patients were eligible (Fig. 1).

### Patient characteristics

At baseline, the median age was 33 years [25–47], 51% female with a mean CD and pfCD duration of 7.0 years (SD 8.7) and 3.7 (SD 4.3) years, respectively (Table 2). Most patients (69%) had chronic symptomatic fistulas [20]. Most of all patients (54%) had only one external orifice (range 1–6). At baseline, the median MAGNIFI-CD score was 18 [9–20] and the mean rVAS was 42.4 (SD 23.4).

**Table 2:** Baseline characteristics

Patient characteristics	
Female, *n* (%)	33 (51)
Age (years) at baseline, median [IQR]	32 [25–47]
BMI (kg/m^2^), median [IQR]	24.6 [21.2–26.6]
Ever smoked, *n* (%)	21 (32)
Disease characteristics	
Age (years) at diagnosis, median [IQR]	25 [20–36]
Montreal disease location, *n* (%): L1, L2, L3, L4, L0^a^	16 (25), 25 (38), 24 (37), 1 (2)
Crohn’s disease duration (years), mean (SD)	7.1 (8.8)
Perianal disease duration (years), mean (SD)	3.8 (4.3)
Perianal fistula classification (TOpClass) according to Geldof et al [20], *n* (%): class 1, 2a, 2b, 2c-i, 2c-ii, 3, 4^b^	2 (3), 30 (46), 15 (23), 4 (6), 10 (15), 4 (6), 0 (0)
External fistula orifices at baseline, *n* (%): 1, 2, ≥ 3 orifices	35 (54), 30 (34), 7 (12)
Previous biological exposure (%)	37 (57)
Previous perianal surgery to attempt fistula closure^c^ (%)	36 (55)
CRP (mg/dL) at baseline, median [IQR]	5.5 [1.5–14.6]
Faecal calprotectin (µg/g) at baseline, median [IQR]	201 [63–1216]
Therapy during study, *n* (%)	
Anti-TNF, mono- or combination therapy	33 (51)
Perianal closure surgery (i.e., LIFT or MAP), with/without biologicals	20 (31)
Hyperbaric oxygen therapy^d^	12 (19)
MRI parameters	
MAGNIFI-CD at baseline, median [IQR]	18 [10–20]
Radiological VAS at baseline, mean (SD)	43 (23)
Predominant feature: granulation tissue at baseline, *n* (%)	43 (66)
Proactive indication for follow-up MRI, *n* (%)	42 (65)
Interval baseline MRI and treatment (months), median [IQR]	3 [1–5]
Interval baseline and follow-up MRI (months), median [IQR]	6 [4–11]
Interval baseline clinical assessment and MRI at baseline (days), median [IQR]	35 [−6 to 81]
Interval clinical assessment at 6 months and MRI at 6 months follow-up (days), mean (95% CI)	9.2 (−10 to 29)

^a^ According to the Montreal classification of disease location, L0: histological confirmed pfCD without prior or current objectified luminal disease

^b^ Perianal fistula classification [20]. Class 1: minimal disease, 2a + b: chronic symptomatic fistulae (for repair or symptom control resp.), 2c-i: early and rapidly progressive disease, 2c-ii: gradually debilitating disease, 3: severe disease with exhausted perineum and adverse features, 4: perianal symptoms after proctectomy

^c^ Perianal closure surgery, i.e., fistulectomy, LIFT, MAP, FILAC

^d^ Add-on hyperbaric oxygen therapy next to biological use

### Responsiveness

#### Clinically relevant change

Among the 65 patients included in this study, 34 (52%) reached short-term (at 6 months) clinical remission (FDA) and 14 (22%) reached short-term clinical response. Twenty-four patients (37%) met the PGA criteria of short-term clinical remission and 23 (35%) clinical response.

The MAGNIFI-CD showed a decrease in responders (*p* = 0.005) and remitters (*p* < 0.001) according to the FDA. A change in the MAGNIFI-CD was seen in non-responders (*p* = 0.22). According to the PGA responders and remitters showed a decrease of the MAGNIFI-CD (both *p* < 0.001), and non-responders showed a constant MAGNIFI-CD over time (*p* = 0.75) (Table 3). Distributions of patients with a decrease and an increase or equal MAGNIFI-CD per clinical outcome are visualised in Supplementary Fig. 2 for FDA and PGA, respectively. Distributions of changes per MAGNIFI-CD feature are displayed in Supplementary Table 2.

**Table 3:** Baseline and post-treatment median MAGNIFI-CD index [IQR] categorised for FDA and PGA

FDA	Baseline	Post-treatment	*p*-value	PGA	Baseline	Post-treatment	*p*-value
Responders (*n* = 14)	20.0 [18.0–22.0]	15.0 [8.5–18.0]	0.005	Responders (*n* = 23)	20.0 [18.0–22.0]	14.0 [9.0–18.0]	< 0.001
Remitters (*n* = 34)	13.5 [7.0–19.3]	5.0 [0.0–14.0]	< 0.001	Remitters (*n* = 24)	11.5 [7.0–17.5]	3.0 [0.0–8.5]	< 0.001
Responders/remitters (*n* = 48)	18.0 [7.5–20.0]	9.0 [0.8–16.0]	< 0.001	Responders/remitters (*n* = 47)	18.0 [7.0–20.0]	9.0 [0.0–16.0]	< 0.001
Non-responders (*n* = 17)	20.0 [12.0–23.0]	18.0 [13.0–21.0]	0.22	Non-responders (*n* = 18)	19.0 [12.5–22.0]	19.0 [14.0–20.5]	0.75

### Radiologically meaningful change

The standardised effect size for MAGNIFI-CD, according to an improvement in the rVAS of one-half of the baseline SD was 1.11 (95% CI 0.68–1.53). Supplementary Table 3 shows the different MAGNIFI-CD scores per time point for patients whose VAS improved one-half of the baseline SD and whose VAS improved less than one-half SD, respectively.

### Correlation with rVAS and biomarkers

The correlations between MAGNIFI-CD and rVAS were strong with a correlation coefficient of 0.86 (95% CI 0.78–0.91, *p* < 0.001) at baseline and very strong with a correlation coefficient of 0.93 (95% CI 0.89–0.96, *p* < 0.001) at follow-up. Change in MAGNIFI-CD index showed a moderate correlation with change in rVAS (ρ = 0.59; 95% CI 0.12–0.25, *p* < 0.001), but negligible correlation with changes in CRP (*n* = 50; ρ = 0.01; 95% CI −0.08 to 0.09, *p* = 0.94) and faecal calprotectin (*n* = 39; ρ = 0.03; 95% CI −0.29 to 0.34, *p* = 0.85).

### Reliability

MAGNIFI-CD index showed an ‘almost perfect’ interobserver agreement with an ICC of 0.87 (95% CI 0.80–0.92). The individual items showed a moderate to substantial interobserver agreement (Table 4). The rVAS showed a substantial agreement with an ICC of 0.78 (95% CI 0.55–0.88).

**Table 4:** Reliability of individual items of MAGNIFI-CD index, total MAGNIFI-CD index and rVAS reported in ICC or weighted Cohen’s κ with 95% confidence interval (95% CI)

Variable	ICC or weighted κ [95% CI]	Agreement^a^
Dominant feature	0.55 [0.41–0.69]	Moderate
T1-hyperintensity	0.69 [0.56–0.83]	Substantial
Extension	0.72 [0.61–0.83]	Substantial
Number of tracts	0.73 [0.62–0.84]	Substantial
Inflammatory mass	0.75 [0.63–0.87]	Substantial
Fistula length	0.77 [0.69–0.86]	Substantial
Total MAGNIFI-CD index	0.87 [0.80–0.92]	Almost perfect
rVAS	0.78 [0.55–0.88]	Substantial

^a^ According to Cohen’s κ interpretation guideline Landis and Koch [18]

### Test accuracy

**Fig. 2:**
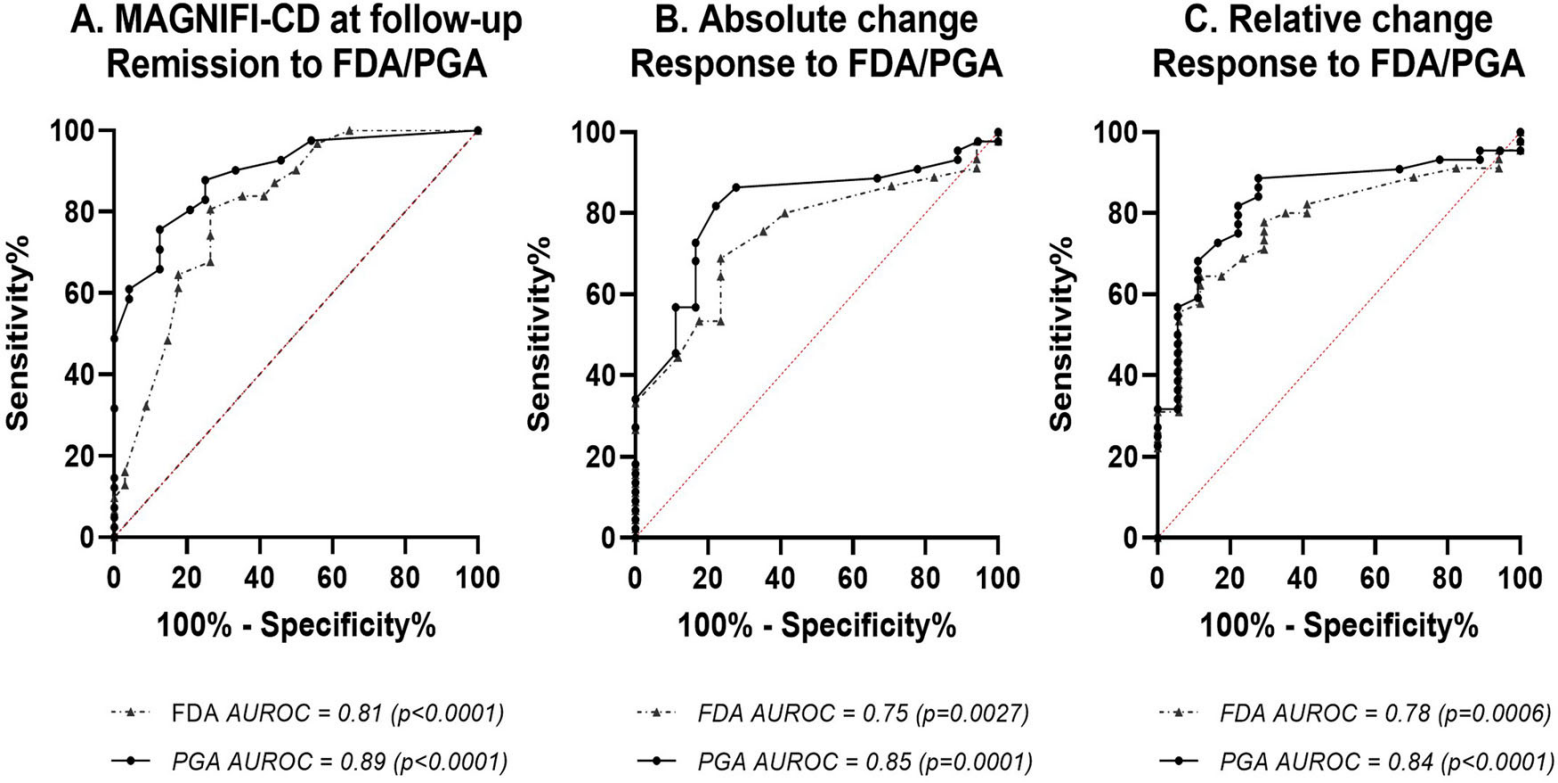
ROC curves of (**A**) MAGNIFI-CD at follow-up classified for remission to FDA and PGA, (**B**) absolute change by response to FDA and PGA and (**C**) relative change by response to FDA and PGA. FDA, fistula drainage assessment; PGA, physician global assessment

The AUROC of MAGNIFI-CD at follow-up classified for clinical remission by FDA and PGA were 0.81 (95% CI 0.70–0.91) and 0.90 (95% CI 0.82–0.97), respectively (Fig. 2). A MAGNIFI-CD at follow-up ≤ 6 was the best discriminant value to define clinical remission (Table 5 and Supplementary Table 4b). A MAGNIFI-CD at follow-up of ≤ 6 discriminated for clinical remission defined by the FDA with a sensitivity of 46% (95% CI 28–65) and specificity of 90% (95% CI 80–100) and defined by PGA with a sensitivity of 63% (95% CI 41–85) and specificity of 90% (95% CI 81–99). Twelve out of the 21 patients meeting the suggested remission cut-off had a MAGNIFI-CD = 0. The other nine patients all scored 3 points for a single unbranched tract, with five patients scoring two additional points for a fistula length between 2.5 and 5 cm (*n* = 3) or granulation tissue as dominant feature (*n* = 2).

**Table 5:** MRI evaluation at 6 months as a function of clinical response and remission by FDA and PGA

	Clinical response by FDA						Clinical response by PGA				
MRI evaluation at 6 months	Pos^a^ (total)	Sens % (95% CI)	Spec % (95% CI)	OR (95% CI)	Accuracy % (95% CI)	*p*-value^b^	Sens % (95% CI)	Spec % (95% CI)	OR (95% CI)	Accuracy % (95% CI)	*p*-value
ΔMAGNIFI-CD ≥ 2	41 (62)	78 (66–90)	65 (42–87)	6.4 (1.9–21.7)	74 (63–85)	0.003	84 (73–95)	78 (59–97)	18.5 (4.7–73.1)	82 (70–91)	< 0.001
ΔMAGNIFI-CD ≥ 25%	32 (62)	64 (50–78)	82 (64–100)	8.4 (2.1–33.9)	69 (65–80)	0.002	68 (54–82)	90 (77–100)	19.3 (3.9–94.8)	74 (62–84)	< 0.001
ΔMAGNIFI-CD ≥ 35%	25 (62)	53 (39–68)	94 (83–100)	18.3 (2.2–149.8)	65 (51–76)	0.001	55 (30–69)	94 (84–100)	20.4 (2.5–167.0)	66 (53–78)	< 0.001

	Clinical remission by FDA						Clinical remission by PGA				
MAGNIFI baseline ≥ 7 and follow-up ≤ 6	16 (59)	46 (28–65)	90 (80–100)	8.1 (2.0–32.9)	69 (56–80)	< 0.001	63 (41–85)	90 (81–99)	15.4 (3.8–62.0)	81 (69–90)	< 0.001
MAGNIFI-CD ≤ 6 or ΔMAGNIFI-CD ≥ 50%^c^	18 (62)	48 (31–66)	90 (80–100)	8.8 (2.2–34.9)	69 (56–80)	< 0.001	67 (47–81)	90 (81–97)	18.5 (4.7–73.1)	82 (70–91)	< 0.001

^a^ Positive test (total *N*)

^b^ *p*-value derived from Pearson χ^2^

^c^ MAGNIFI-CD at follow-up ≤ 6 and baseline ≥ 7

The AUROC of absolute change and relative change of MAGNIFI-CD over time classified for clinical response by FDA were 0.74 (95% CI 0.62–0.87) and 0.78 (95% CI 0.67–0.90), and classified for clinical response by PGA 0.82 (95% CI 0.70–0.93) for absolute change and 0.84 (95% CI 0.73–0.94) for relative change, respectively (Fig. 2).

A decrease of two points or 25% appeared to be the best discriminant value to define clinical response (Table 5 and Supplementary Table 4a). The combination of a decrease of 2 points and 25% did not result in better test accuracy. A decrease of 2 points defined clinical response according to FDA with 78% (95% CI 66–90) sensitivity and 65% (95% CI 42–87) specificity and defined by PGA with 84% (95% CI 73–95) sensitivity and 78% (95% CI 59–97) specificity. A decrease of 25% defined clinical response by FDA with 64% (95% CI 50–78) sensitivity and 82% (95% CI 64–100) specificity and defined by PGA with 68% (95% CI 54–82) sensitivity and 90% (95% CI 77–100) specificity.

### Long-term follow-up

All patients (*n* = 20) that met the suggested remission cut-off (follow-up MAGNIFI-CD ≤ 6) had no re-intervention at 12 months after the follow-up MRI, compared to 34 of 45 patients (76%) that did not meet these criteria (*p* = 0.015). The suggested response cut-off (MAGNIFI-CD decrease of ≥ 2 points) showed no significant difference in re-interventions at 12 months after the follow-up MRI, respectively 7/41 (17%) and 4/24 (17%) of patients with and without a MAGNIFI-CD decrease of ≥ 2 points had a re-intervention within 12 months after the follow-up MRI (*p* = 0.97).

Twelve months after follow-up MRI, eleven patients (17%) underwent a surgical re-intervention (five seton replacements, two new abscesses, two new fistulas, one defunctioning ostomy and one proctectomy) after a mean of 5.3 months (SD 4.7), no medical re-interventions (i.e., switching biologicals) were done for 18 months follow-up. Twelve months after follow-up MRI, 10 patients (15%) and 16 patients (25%) achieved long-term response by FDA and PGA, respectively, and 40 (62%) and 30 patients (46%) achieved long-term remission by FDA and PGA, respectively.

## Discussion

In this study, we externally validated the MAGNIFI-CD index on responsiveness, reliability, test accuracy and defined cut-offs of the MAGNIFI-CD index for clinically meaningful outcomes. The MAGNIFI-CD index was responsive in clinical responders and remitters according to both the FDA and PGA. It also showed a reproducible standardised effect size (based on a predefined meaningful statistical change defined as an improvement in the rVAS of one-half of the baseline standard deviation) compared to Hindryckx et al [12]. Furthermore, the total index exhibited an ‘almost perfect’ interobserver agreement underlining a good reliability for research practice. A MAGNIFI-CD ≤ 6 is indicative of clinical remission at 6 months and re-intervention-free survival at 18 months. A decrease of 2 points in the MAGNIFI-CD can serve as a cut-off for short-term clinical improvement.

Robust responsiveness to clinically relevant change was shown with both FDA and PGA. However, approximately 20% of clinical responders and remitters showed an unchanged or increased MAGNIFI-CD score over time. Explanations for this occurrence are at first that we have chosen to read the MRIs in random order and blinded for clinical time point, which implies that some subtle changes are not picked up. Another reason is that the MAGNIFI-CD index consists of different items and the worst present feature of each item is scored; while sometimes the overall disease activity is decreasing, this is not captured by the score. However, this is lower compared to earlier reported data using the modified Van Assche score where 38% of the clinical responders had equal or increased modified Van Assche score [21]. In that study, the definition of response was absent or reduced fistula drainage and/or a reduction in external openings. Whilst, in our present study the FDA and the PGA were used. Importantly, the addition of T1-hyperintensity and fistula length to the MAGNIFI-CD index leads to more responsiveness of the index—which showed a decrease over time of approximately 40% of the responders and remitters for both variables.

The MAGNIFI-CD showed a moderate correlation between MAGNIFI-CD and rVAS and a negligible correlation with CRP and faecal calprotectin, data which are comparable to those of Hindryckx et al [12]. As previously shown, traditional biomarkers used in Crohn’s disease are less sensitive to change in pfCD [12, 22].

Our study showed an ‘almost perfect’ interobserver reliability for the MAGNIFI-CD index. Hindryckx et al reported a lower interobserver reliability (ICC = 0.74), most likely related to the higher number (four) of reading radiologists [12]. Despite a session for the reading radiologists in which definitions were clarified and items were trained using test cases, the item “dominant feature” was consistently the poorest performer. An explanation may be that the cut-off value of > 50% fluid or pus, granulation tissue or fibrosis is difficult to visualise in combination with this feature being unique in that radiologists need not assess the worst case, but rather the most prevalent one (the largest proportion). As an alternative, a more pragmatic approach would be to assess “the most prevalent feature” as this could lead to better understanding for the scoring radiologists. Since previous literature reported almost perfect intra-observer reliability [12], we did not repeat this analysis.

We assessed different cut-offs for the MAGNIFI-CD to define clinically relevant change. Results showed that the MAGNIFI-CD is specific for response (a decrease of 2 points) and remission (MAGNIFI-CD ≤ 6) both at 6 months and the latter showed a prolonged clinical benefit at 18 months. The MAGNIFI-CD showed predictive value as a post-treatment MAGNIFI-CD ≤ 6 predicted long-term clinical closure at 12 months with a specificity of 91% and sensitivity of 87% in a post-hoc analysis of the PISA-II trial [15]. Compared to our cohort, van Rijn et al [15] reported higher sensitivity rates, this is very likely caused by timing of the post-treatment MRI (6 vs 18 months) and the selection the binary classifier imposes. Gentle finger compression is highly sensitive and less specific in detecting fistula response and remission, especially at a relatively early time point, whereas the addition of re-interventions or patient-reported symptoms will increase the specificity. The nature of some individual items of the MAGNIFI-CD characterises disease severity or the degree of pelvic sepsis rather than fistula activity, potentially impairing sensitivity to capture therapy response. Examples are the items ‘inflammatory mass’ (especially the collections) and ‘extension.’ The addition of a reduction of 50% of the baseline MAGNIFI-CD to the remission criteria increases the sensitivity and could be beneficial towards this definition at 6 months, especially in treatments with a mode of action that requires more time. Patients meeting the suggested remission cut-off in a majority had a MAGNIFI-CD at follow-up of 0 or scored 3 points for a single tract and either 2 points for active fistula length or granulation tissue. No patients meeting a MAGNIFI-CD ≤ 6 had medical or surgical re-interventions 12 months after the follow-up MRI.

A decrease of 2 points of the MAGNIFI-CD over time seems to be most appropriate to define response at 6 months. Although a relative decrease of 25% has similar test ability properties, the advantage of a relative decrease in lower baseline scores is limited considering the remission cut-off (MAGNIFI-CD ≤ 6). The predictive value of this cut-off value is limited regarding long-term re-intervention-free rates, a result of the lack of clinical relevance that is imposed by the underlying criteria for clinical response. However, an objective definition of therapy response may guide in establishing a dose-response relationship in research practice and therapy intensification in clinical practice.

It should be mentioned that the evaluation of an MRI based score to determine fistula healing is extremely challenging. Currently, no reference standard for radiological nor well-validated clinical outcomes is available as comparison. During the development of the MAGNIFI-CD index, improvement of a radiological VAS (0–100 mm) of one-half of the baseline standard deviation was used as the reference standard for response assessment. One can argue this method, especially because the rVAS for pfCD is subjective—since the radiologist assigns a score between 0 and 100 for total disease activity—and has not been validated. The moderate correlation between change in MAGNIFI-CD and change in rVAS in the original development paper and our cohort, and the absence of other adequate reference standards justifies this choice.

The most important limitation of our study is the retrospective nature of this cohort and subjective nature and not validated clinical response standards (i.e., FDA). The absence of two consecutive MRIs, prolonged MRI interval and incomplete MRI protocols due to missing T1-weighted post-contrast images hampered our sample size. All patients were treated in tertiary IBD clinics which implies a bias towards a patient cohort with more complex disease. In contrast to clinical practice, we used a blinded (for clinical information and scan order) and randomised assessment of all MRIs as an established, rigorous methodology to evaluate test responsiveness. This method is a strength of our study and minimises bias, which is often a prerequisite for the use of a test in clinical trials. Although interobserver reliability was high, our method could hamper the identification of most subtle changes in disease activity in contrast to the parallel assessment of baseline and follow-up MRIs as is common in clinical practice.

However, the MAGNIFI-CD has predefined criteria and major relevant changes will lead to a different score with either approach. Minor changes may stay unnoticed at the blinded read, but in most situations, these do not impact the MAGNIFI-CD score, and if so, this impact will be small.

This study shows the external validation of the MAGNIFI-CD in a patient population with different surgical and medical treatments, whilst developed in a specific trial population (ADMIRE-CD), which often does not overlap with clinical practice. The analyses were strengthened by blinded MRI scoring by multiple readers, and the use of different clinical and radiological outcome parameters to robustly assess the MAGNIFI-CD index. The MAGNIFI-CD is a well-structured, responsive scoring instrument to assess fistula severity and activity that allows to quantitatively detect changes in therapy response.

## Conclusions

The MAGNIFI-CD index shows good external validity with clinically relevant responsiveness and almost perfect interobserver agreement. Optimal radiological cut-offs for remission (6 months MAGNIFI-CD ≤ 6) and response (decrease in 2 points MAGNIFI-CD) showed to be indicative for objective treatment monitoring in pfCD.

## Supplementary information

ELECTRONIC SUPPLEMENTARY MATERIAL

## Data Availability

The data underlying this article will be shared on reasonable request to the corresponding author.

## Abbreviations

CD: Crohn’s disease
FDA: Fistula drainage assessment
FS: Fat suppression
HBO: Hyperbaric oxygen therapy
MAGNIFI-CD: Magnetic resonance novel index for fistula imaging in CD
pfCD: Perianal fistulising Crohn’s disease
PGA: Physician global assessment
rVAS: Radiological Visual Analogue Scale
