# Supplementary material for: External validation of the MAGNIFI-CD index in patients with complex perianal fistulising Crohn’s disease
Source: Eur Radiol. 2024 Aug 30;35(3):1428–39. doi: 10.1007/s00330-024-11029-3 (PMC11836172; doi:10.1007/s00330-024-11029-3)
Supplement: Supplementary file 1 — ELECTRONIC SUPPLEMENTARY MATERIAL [file 330_2024_11029_MOESM1_ESM.pdf]

External Validation of the MAGNIFI-CD Index in Patients with Complex Perianal Fistulising Crohn's Disease

ELECTRONIC SUPPLEMENTARY MATERIAL

Eur Radiol (2024) Beek KJ, Mulders LGM, van Rijn KL, et al.

Supplementary figure 1. Scaling examples (axial T1-weighted images with fat suppression after administration of gadolinium-based contrast agent) of the radiological Visual Analogue Scale (rVAS) with a range of 0 mm – 100 mm.

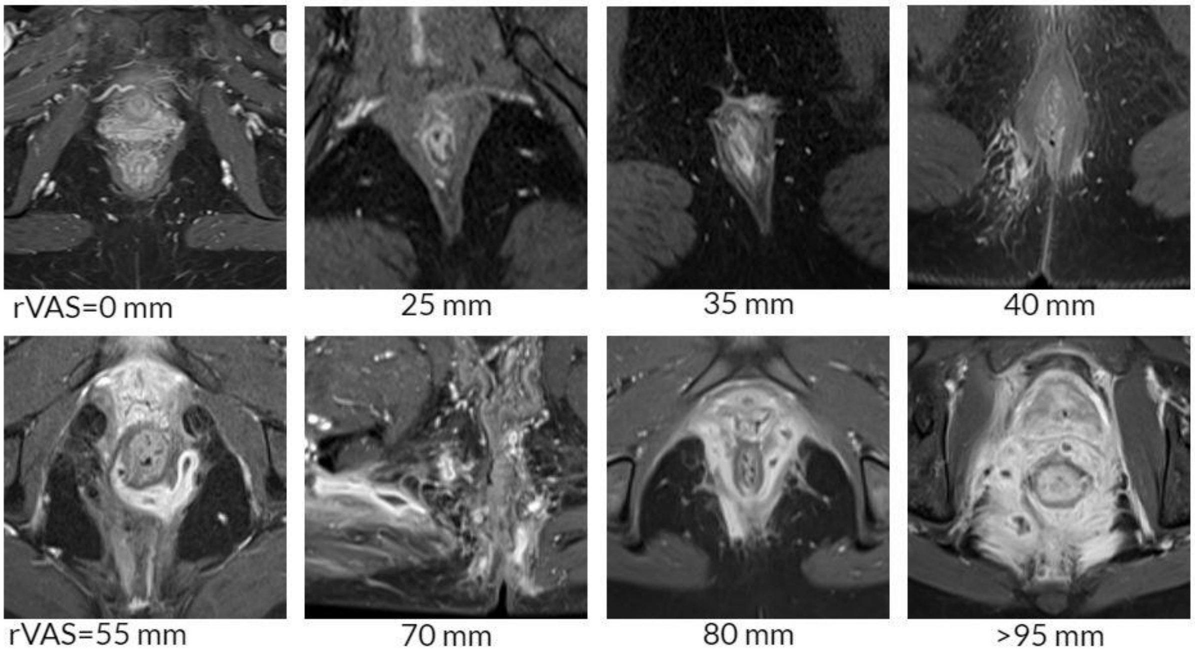

Supplementary figure 2a and b. Distribution of patients with decrease of MAGNIFI-CD over time and equal or increased MAGNIFI-CD per clinical outcome

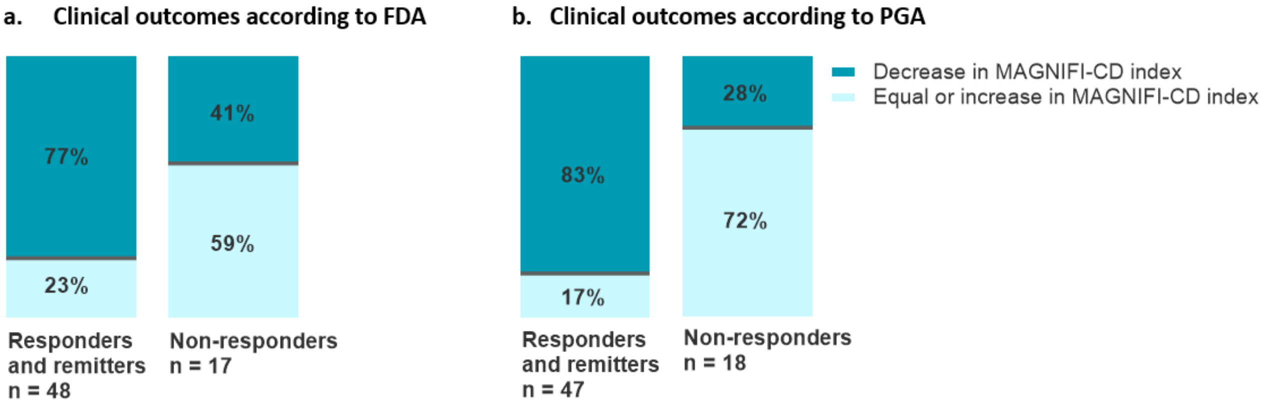

In total 11 cases which showed an equal or increased MAGNIFI-CD over time were clinical responders or remitters according to the FDA. Three patients were classified as "non-responders" according to the PGA. For the remaining eight patients with the equal or increase in the MAGNIFI-CD score could be explained by two factors i) radiological scoring was blinded for time point ii) subtle changes are not captured by the MAGNIFI-CD index, since the items are scored based on the feature that is the worst.

Supplementary table 1. Definitions fistula drainage assessment (FDA) and physician global assessment (PGA)

**Fistula drainage assessment (14)**	**Definition**
Remission	100% decrease in the number of draining external fistula openings upon gentle finger compression during consecutive visits compared to baseline
Response	50% decrease in the number of draining external fistula openings upon gentle finger compression during consecutive visits compared to baseline
Non-response	No decrease in the number of draining external fistula openings upon gentle finger compression during consecutive visits compared to baseline
**Physician global assessment**	
Remission	Remission was defined as an absence of patient reported symptoms (discharge, pain, swelling), absence of draining fistula openings upon gentle finger compression by the treating physician (14), absence of clinically assessed abscesses and no surgical re-interventions or changes in medical treatment related to the fistula since baseline evaluation
Response	Response was defined as improvement of above-mentioned patient reported symptoms or the number of draining fistulas upon gentle finger compression
Non-response	Non-response was defined as no improvement of patient symptoms or intercurrent need for surgical or medical intervention (i.e., switching biologicals, seton placement, abscess drainage, ostomy, proctectomy)

Supplementary table 2. Responsiveness per MAGNIFI-CD parameter for clinical responders/remitters and non-responders

**FDA**	**Responders/remitters N=48**	**Non-responders N=17**	**PGA**	**Responders/remitters N=47**	**Non-responders N=18**
**Number of fistula**					
**tracts**	16 (33%)	1 (6%)		16 (34%)	1 (6%)
**Decreased, n (%)**	30 (63%)	15 (88%)		29 (62%)	16 (88%)
**Unchanged, n (%)**	2 (4%)	1 (6%)		2 (4%)	1 (6%)
**Increased, n (%)**					
**T1 hyperintensity**					
	20 (42%)	-		20 (43%)	-
	26 (54%)	17 (100%)		25 (52%)	18 (100%)
	2 (4%)	-		2 (4%)	-
**Predominant feature**					
	25 (52%)	4 (24%)		27 (58%)	2 (11%)
	19 (40%)	12 (71%)		17 (36%)	14 (78%)
	4 (8%)	1 (6%)		3 (6%)	2 (11%)
**Fistula length**					
	19 (40%)	1 (6%)		18 (38%)	2 (11%)
	28 (58%)	15 (88%)		28 (60%)	15 (83%)
	1 (2%)	1 (6%)		1 (2%)	1 (6%)
**Extension**					
	11 (23%)	-		11 (23%)	-
	37 (77%)	16 (94%)		36 (77%)	17 (94%)
	-	1 (6%)		-	1 (6%)
**Inflammatory mass**					
	17 (36%)	5 (29%)		18 (38%)	4 (22%)
	27 (56%)	9 (53%)		27 (57%)	9 (50%)
	4 (8%)	3 (18%)		2 (4%)	5 (28%)

Supplementary table 3. MAGNIFI-CD change over time based on radiologically meaningful change (in Hindryckx indicated as predefined statistical meaningful change)

	**Mean (±SD) and median [IQR] MAGNIFI-CD index**	
	Baseline	Follow-up
**rVAS improvement one-half of baseline SD (n=35)**	17.1 (±6.3)	10.4 (±7.9)
	20.0 [12.0-22.0]	14.0 [3.0-18.0]
**rVAS improvement of less than one-half of baseline SD (n=20)**	13.7 (±7.0)	12.0 (±7.8)
	17.0 [7.0-19.0]	10.5 [6.5-20.0]

Supplementary table 4a. Test characteristics of MAGNIFI-CD at follow-up, change and relative change discriminating non response versus response and remission.

			Clinical response according to FDA								Clinical response according to PGA							
**MAGNIFI-CD**	Total	POS	Sens	Spec	PPV	NPV	YI	OR	Accuracy	P-value	Sens	Spec	PPV	NPV	YI	OR	Accuracy	P-value
Baseline > 15 + FU ≤ 15	39	15	54	100	100	46	0.54	n.a.	67	0.006	56	100	100	50	0.56	n.a.	69	0.002
Baseline > 14 + FU ≤ 14	40	15	54	100	100	48	0.54	n.a.	68	0.003	56	100	100	52	0.56	n.a.	70	<0.001
Baseline > 13 + FU ≤ 13	42	13	43	100	100	41	0.43	n.a.	60	0.016	45	100	100	45	0.45	n.a.	62	0.008
Baseline > 12 + FU ≤ 12	44	14	45	100	100	43	0.45	n.a.	61	0.006	47	100	100	47	0.47	n.a.	64	0.003
Baseline > 11 + FU ≤ 11	46	14	42	100	100	41	0.42	9	59	0.013	44	100	100	44	0.44	n.a.	61	0.007
Baseline > 10 + FU ≤ 10	48	14	39	93	93	41	0.33	3	56	0.015	41	94	93	44	0.34	10	58	0.008
Baseline > 9 + FU ≤ 9	50	12	30	88	83	39	0.19	4	50	0.009	31	89	83	42	0.20	4	52	0.005
Baseline > 8 + FU ≤ 8	54	15	35	88	87	38	0.23	8	52	0.016	36	89	87	41	0.25	5	54	0.01
Baseline > 7 + FU ≤ 7	54	13	32	94	92	39	0.27	9	52	0.008	33	94	92	41	0.28	9	54	0.005
Baseline > 6 + FU ≤ 6	59	16	36	94	94	37	0.30	9	53	0.017	37	94	94	40	0.31	10	54	0.011
Baseline > 5 + FU ≤ 5	59	16	36	94	94	37	0.30	6	53	0.017	37	94	94	40	0.31	10	54	0.011
Baseline > 4 + FU ≤ 4	61	13	27	94	92	33	0.21	6	46	0.082	28	94	92	35	0.22	7	48	0.062
Baseline > 3 + FU ≤ 3	61	13	27	94	92	33	0.21	n.a.	46	0.082	28	94	92	35	0.22	7	48	0.062
Baseline > 2 + FU ≤ 2	62	10	22	100	100	33	0.22	n.a.	44	0.056	23	100	100	35	0.23	n.a.	45	0.045
Baseline > 1 + FU ≤ 1	62	10	22	100	100	33	0.22	n.a.	44	0.056	23	100	100	35	0.23	n.a.	45	0.045
FU = 0	65	12	24	100	100	32	0.25	n.a.	45	0.022	26	100	100	34	0.26	n.a.	46	0.018
ΔMAGNIFI-CD ≥1	62	44	82	59	84	56	0.41	7	76	0.003	89	72	89	72	0.61	20	84	<0.001
ΔMAGNIFI-CD ≥2	62	41	78	65	85	52	0.42	6	74	0.003	84	78	90	67	0.62	19	82	<0.001
ΔMAGNIFI-CD ≥3	62	36	71	76	89	50	0.48	8	73	0.002	75	83	92	58	0.58	15	77	<0.001
ΔMAGNIFI-CD ≥4	61	34	68	76	88	48	0.45	7	70	0.003	72	83	91	56	0.55	13	75	<0.001
ΔMAGNIFI-CD ≥5	61	29	57	76	86	41	0.33	4	62	0.028	60	83	90	47	0.44	8	67	0.003
ΔMAGNIFI-CD ≥6	59	28	60	82	89	45	0.42	7	66	0.003	63	89	93	52	0.52	14	71	<0.001
ΔMAGNIFI-CD ≥7	59	23	50	88	91	42	0.38	n.a.	61	0.006	51	89	91	44	0.40	8	63	0.003

ΔMAGNIFI-CD ≥8	54	16	43	100	100	45	0.43	n.a.	61	<0.001	44	100	100	47	0.44	n.a.	63	<0.001
ΔMAGNIFI-CD ≥9	54	13	35	100	100	41	0.35	n.a.	56	0.001	36	100	100	44	0.36	n.a.	57	<0.001
ΔMAGNIFI-CD ≥10	50	8	24	100	100	40	0.24	n.a.	50	0.027	25	100	100	43	0.25	n.a.	52	0.021
ΔMAGNIFI-CD ≥20%	62	37	71	71	86	48	0.42	6	71	0.002	75	78	89	56	0.53	11	76	<0.001
ΔMAGNIFI-CD ≥25%	62	32	64	82	91	47	0.47	8	69	0.002	68	89	94	53	0.57	17	74	<0.001
ΔMAGNIFI-CD ≥30%	62	28	58	88	93	44	0.46	10	66	0.001	59	89	93	47	0.48	12	68	0.001
ΔMAGNIFI-CD ≥35%	62	25	53	94	96	43	0.47	18	65	0.012	55	94	96	46	0.49	20	66	<0.001
ΔMAGNIFI-CD ≥40%	62	20	42	94	95	38	0.36	12	56	0.025	43	94	95	40	0.38	13	58	0.008
ΔMAGNIFI-CD ≥45%	62	18	38	94	94	36	0.32	10	53	0.034	39	94	94	39	0.33	11	55	0.017
ΔMAGNIFI-CD ≥50%	62	17	36	94	94	36	0.30	9	52	0.047	36	94	94	38	0.31	10	53	0.024
ΔMAGNIFI-CD ≥55%	62	16	33	94	94	35	0.27	8	50	0.063	34	94	94	37	0.29	9	52	0.033
ΔMAGNIFI-CD ≥60%	62	15	31	94	93	34	0.25	7	48	0.063	32	94	93	36	0.26	8	50	0.046
ΔMAGNIFI-CD ≥65%	62	15	31	94	93	34	0.25	7	48	0.063	32	94	93	36	0.26	8	50	0.046
ΔMAGNIFI-CD ≥70%	62	15	31	94	93	34	0.25	7	48	0.017	32	94	93	36	0.26	8	50	0.046
ΔMAGNIFI-CD ≥75%	62	14	31	100	100	35	0.31	n.a.	50	0.042	32	100	100	38	0.32	n.a.	52	0.012
ΔMAGNIFI-CD ≥80%	62	11	24	100	100	33	0.24	n.a.	45	0.042	25	100	100	35	0.25	n.a.	47	0.033
ΔMAGNIFI-CD ≥85%	62	11	24	100	100	33	0.24	n.a.	45	0.056	25	100	100	35	0.25	n.a.	47	0.033
ΔMAGNIFI-CD ≥90%	62	10	22	100	100	33	0.22	n.a.	44	0.056	23	100	100	35	0.23	n.a.	45	0.045
ΔMAGNIFI-CD ≥95%	62	10	22	100	100	33	0.22	n.a.	44	0.056	23	100	100	35	0.23	n.a.	45	0.045
ΔMAGNIFI-CD 100%	62	10	22	100	100	33	0.22	n.a.	44	0.006	23	100	100	35	0.23	n.a.	45	0.045
ΔMAGNIFI-CD≥2OR≥25%	62	41	78	65	85	52	0.42	62	74	0.002	84	78	90	67	0.62	19	82	0.002
ΔMAGNIFI-CD≥2OR≥35%	62	41	78	65	85	52	0.42	62	74	0.002	84	78	90	67	0.62	19	82	0.002

Supplementary table 4b. Test characteristics of MAGNIFI-CD at follow-up, change and relative change with binary classifier clinical non-response and response versus remission by FDA and PGA

MAGNIFI-CD	Total	POS	Clinical remission according to FDA								Clinical remission according to PGA							
			Sens	Spec	PPV	NPV	YI	OR	Accuracy	P-value	Sens	Spec	PPV	NPV	YI	OR	Accuracy	P-value
Baseline > 15 + FU ≤ 15	39	15	63	78	67	75	0.41	6	72	<0.001	88	74	47	96	0.62	20	77	0.002
Baseline > 14 + FU ≤ 14	40	15	63	79	67	76	0.42	6	73	0.003	88	75	47	96	0.63	21	78	<0.001
Baseline > 13 + FU ≤ 13	42	13	50	83	69	69	0.33	5	69	0.001	70	81	54	90	0.51	10	79	<0.001
Baseline > 12 + FU ≤ 12	44	14	53	84	71	70	0.37	6	70	0.009	73	82	57	90	0.55	12	80	<0.001
Baseline > 11 + FU ≤ 11	46	14	57	92	86	72	0.49	15	76	0.004	77	88	71	91	0.65	24	85	<0.001
Baseline > 10 + FU ≤ 10	48	14	57	93	86	74	0.50	17	77	<0.001	77	89	71	91	0.65	26	85	<0.001
Baseline > 9 + FU ≤ 9	50	12	43	90	75	68	0.33	7	70	<0.001	62	89	67	87	0.51	13	82	<0.001
Baseline > 8 + FU ≤ 8	54	15	48	90	80	67	0.38	8	70	<0.001	60	85	60	85	0.45	8	78	<0.001
Baseline > 7 + FU ≤ 7	54	13	44	93	85	66	0.37	11	70	<0.001	60	90	69	85	0.50	13	81	<0.001
Baseline > 6 + FU ≤ 6	59	16	46	90	81	65	0.37	8	69	<0.001	63	90	75	84	0.53	15	81	<0.001
Baseline > 5 + FU ≤ 5	59	16	46	90	81	65	0.37	8	69	<0.001	63	90	75	84	0.53	15	81	<0.001
Baseline > 4 + FU ≤ 4	61	13	40	97	92	63	0.37	20	69	<0.001	50	93	77	79	0.43	13	79	<0.001
Baseline > 3 + FU ≤ 3	61	13	40	97	92	63	0.37	20	69	<0.001	50	93	77	79	0.43	13	79	<0.001
Baseline > 2 + FU ≤ 2	62	10	32	100	100	60	0.32	n.a.	66	<0.001	43	98	90	77	0.40	30	79	<0.001
Baseline > 1 + FU ≤ 1	62	10	32	100	100	60	0.32	n.a.	66	<0.001	43	98	90	77	0.40	30	79	<0.001
FU = 0	65	12	35	100	100	58	0.35	n.a.	66	<0.001	46	98	92	75	0.43	34	78	<0.001
ΔMAGNIFI-CD ≥1	62	44	84	42	59	72	0.26	4	63	0.019	95	41	45	94	0.37	14	60	<0.001
ΔMAGNIFI-CD ≥2	62	41	77	45	59	67	0.23	3	61	0.041	95	49	49	95	0.44	19	65	<0.001
ΔMAGNIFI-CD ≥3	62	36	74	58	64	69	0.32	4	66	0.009	90	59	53	92	0.49	13	69	<0.001
ΔMAGNIFI-CD ≥4	61	34	73	61	65	70	0.35	4	67	0.004	90	61	53	93	0.51	14	70	<0.001
ΔMAGNIFI-CD ≥5	61	29	57	61	59	59	0.18	2	59	0.053	65	61	45	78	0.26	3	62	0.005
ΔMAGNIFI-CD ≥6	59	28	61	65	61	65	0.25	3	63	0.008	68	63	46	81	0.31	4	64	0.005
ΔMAGNIFI-CD ≥7	59	23	50	71	61	61	0.21	2	61	0.013	63	73	52	81	0.36	5	69	0.002
ΔMAGNIFI-CD ≥8	54	16	44	83	69	63	0.27	4	65	0.01	60	82	56	84	0.42	7	76	<0.001
ΔMAGNIFI-CD ≥9	54	13	44	93	85	66	0.37	11	70	<0.001	60	90	69	85	0.50	13	81	<0.001

ΔMAGNIFI-CD ≥10	50	8	33	97	88	67	0.30	14	70	0.004	46	95	75	83	0.41	15	82	<0.001
ΔMAGNIFI-CD ≥20%	62	37	74	55	62	68	0.29	3	65	0.016	95	59	54	96	0.54	28	71	<0.001
ΔMAGNIFI-CD ≥25%	62	32	65	61	63	63%	0.26	3	63	0.03	81	65	53	88	0.46	8	70	<0.001
ΔMAGNIFI-CD ≥30%	62	28	61	71	68	65	0.32	4	66	0.009	71	68	54	82	0.40	5	69	<0.001
ΔMAGNIFI-CD ≥35%	62	25	58	77	72	65	0.35	5	68	0.004	71	76	60	84	0.47	8	74	<0.001
ΔMAGNIFI-CD ≥40%	62	20	55	90	85	67	0.45	11	73	<0.001	71	88	75	86	0.59	18	82	<0.001
ΔMAGNIFI-CD ≥45%	62	18	52	94	89	66	0.45	15	73	<0.001	67	90	78	84	0.57	19	82	<0.001
ΔMAGNIFI-CD ≥50%	62	17	48	94	88	64	0.42	14	71	<0.001	62	90	76	82	0.52	15	81	<0.001
ΔMAGNIFI-CD ≥55%	62	16	45	94	88	63	0.39	12	69	<0.001	57	90	75	80	0.47	12	79	<0.001
ΔMAGNIFI-CD ≥60%	62	15	42	94	87	62	0.35	10	68	0.001	52	90	73	79	0.43	10	77	<0.001
ΔMAGNIFI-CD ≥65%	62	15	42	94	87	62	0.35	10	68	0.001	52	90	73	79	0.43	10	77	<0.001
ΔMAGNIFI-CD ≥70%	62	15	42	94	87	62	0.35	10	68	0.001	52	90	73	79	0.43	10	77	<0.001
ΔMAGNIFI-CD ≥75%	62	14	42	97	93	63	0.39	22	69	<0.001	52	93	79	79	0.45	14	79	<0.001
ΔMAGNIFI-CD ≥80%	62	11	35	100	100	61	0.35	n.a.	68	<0.001	43	95	82	76	0.38	15	77	<0.001
ΔMAGNIFI-CD ≥85%	62	11	35	100	100	61	0.35	n.a.	68	<0.001	43	95	82	76	0.38	15	77	<0.001
ΔMAGNIFI-CD ≥90%	62	10	32	100	100	60	0.32	n.a.	66	<0.001	43	98	90	77	0.40	30	79	<0.001
ΔMAGNIFI-CD ≥95%	62	10	32	100	100	60	0.32	n.a.	66	<0.001	43	98	90	77	0.40	30	79	<0.001
ΔMAGNIFI-CD 100%	62	10	32	100	100	60	0.32	n.a.	66	<0.001	43	98	90	77	0.40	30	79	<0.001
MAGNIFI-CD≤6ORΔ≥50%	62	41	48	90	83	64	0.39	9	69	<0.001	67	90	78	84	0.57	19	82	<0.001
